# A Single Subject, Feasibility Study of Using a Non-Contact Measurement to “Visualize” Temperature at Body-Seat Interface

**DOI:** 10.3390/s22103941

**Published:** 2022-05-23

**Authors:** Zhuofu Liu, Vincenzo Cascioli, Peter W. McCarthy

**Affiliations:** 1 The Higher Educational Key Laboratory for Measuring and Control Technology and Instrumentations of Heilongjiang Province, Harbin University of Science and Technology, Harbin 150080, China; 2 Murdoch University Chiropractic Clinic, Murdoch University, Murdoch 6150, Australia; v.cascioli@murdoch.edu.au; 3 Faculty of Life Science and Education, University of South Wales, Treforest, Pontypridd CF37 1DL, UK; peter.mccarthy@southwales.ac.uk; 4 Faculty of Health Sciences, Durban University of Technology, Durban 1334, South Africa

**Keywords:** temperature measurement, sit, body-seat interface, contact and non-contact, infrared sensor

## Abstract

Measuring temperature changes at the body-seat interface has been drawing increased attention from both industrial and scientific fields, due to the increasingly sedentary nature from daily leisure activity to routine work. Although contact measurement is considered the gold standard, it can affect the local micro-environment and the perception of sitting comfort. A non-contact temperature measurement system was developed to determine the interface temperature using data gathered unobtrusively and continuously from an infrared sensor (IRs). System performance was evaluated regarding linearity, hysteresis, reliability and accuracy. Then a healthy participant sat for an hour on low/intermediate density foams with thickness varying from 0.5–8 cm while body-seat interface temperature was measured simultaneously using a temperature sensor (contact) and an IRs (non-contact). IRs data were filtered with empirical mode decomposition and fractal scaling indices before a data-driven artificial neural network was utilized to estimate the contact surface temperature. A strong correlation existed between non-contact and contact temperature measurement (ρ > 0.85) and the estimation results showed a low root mean square error (RMSE) (<0.07 for low density foam and <0.16 for intermediate density foam) and high Nash-Sutcliff efficiency (NSE) values (≈1 for both types of foam materials).

## 1. Introduction

People engaged in prolonged sitting (e.g., wheelchair users) are at risk of developing problems that can be exacerbated by excessive temperature building up at the user-seat interface. One such issue is that of epidermal ulceration, which is a very painful illness and can lead to life-threatening complications, especially for people who suffer from impaired sensation, poor circulation or are unable to move by themselves [1,2,3]. Skin ulcer formation is very complicated. However, unobserved thermal accumulation at the body-seat interface has a strong bearing on skin tissue integrity [4,5,6], by increasing metabolic demand at a time when there is a decreased blood supply due to tissue compression [7,8]. As a result of the above, friction and shear force are more prone to cause tissue damage, eventually resulting in a breach in the integrity of the skin (ulceration) [9,10]. In males, a temperature increase at the body-seat interface can additionally lead to the deterioration of semen quality and quantity as well as sperm chromatin structure [11]. A scrotal temperature increase of up to 3 °C has been reported following sitting on an office chair for as little as 20-min [12].

The current method of maintaining tissue health and integrity of patients, or those requiring care in a care home environment, requires care workers or nurses to physically check at-risk people regularly [13,14]. This can be both time consuming and, in some groups, often met with its own challenges (e.g., disturbing patients with dementia, personal space invasion) and may not even be necessary. Therefore, studying thermal changes at the body-seat interface could be an effective means to enable both carers and nurses to intervene only when necessary and, most importantly, only when appropriate. Such study could help reduce the likelihood of developing temperature related problems (e.g., pressure ulcer formation) as well as preventing unnecessary disruption and personal space invasion for the person under care, potentially also enhancing their seating comfort. Published research methods regarding thermal characteristics of the micro-environment between the body and the support surface can be classified into two categories based on their methodology: subjective and objective (Table 1).

Although questionnaires are widely used to evaluate sitting comfort, the results are subject to participant bias [15]. In addition, some perceptions are difficult to exactly delineate, such as thermal comfort or discomfort of a particular body part, unless extreme. As a result, such studies are likely to require large populations to be investigated over prolonged durations in order to arrive at a firm conclusion [10]. To overcome the inherent drawbacks of subjective evaluations, researchers have increasingly focused on the applicability of objective methods.

Although objective and reliable monitoring of thermal changes at the interface between the skin surface and the seat is important, this has previously necessitated the use of temperature probes either mounted on the seat cushion or taped directly onto the skin [13,16], neither of which could be considered ideal. When sensors are attached directly to the body [16] they are prone to damage (e.g., twisted wire or broken solder joints) as well as being perceptible, thus affecting the subject’s perception of comfort [10]. Furthermore, they can create a local micro-environment which is independent of the surrounding skin, not being subject to evaporation opportunities on movement and potentially raising local temperatures by adding to any insulation locally. Indeed, even embedding sensors in a seat cushion may both be perceptible and directly affect comfort. Apart from affecting integrity of the seating material, such placement in a clinical setting would also make patients vulnerable to damage the sensor (including its circuitry) or even potential electrical shock due to water spillage or urine leakage. Although objectively measuring thermal changes at the body-seat contact surface appears important for reducing sedentary-related skin issues (e.g., pressure ulcers) [2,10], measurement should be performed away from those regions that are most susceptible to ulceration. As these regions are usually around bony prominences, using rigid temperature detectors would only be expected to make the situation worse (trapping skin between the bone and the rigid sensor will increase local pressure and more quickly lead to ischemia or extreme discomfort, consequently negating any benefit of the seat material being studied [10]). Advantages of non-contact temperature measurements could include being less obtrusive to the subject/patient, easier to be discretely embedded into the chair frame, decreased likelihood of disturbing the sensor or its circuitry resulting from sitting and fidgeting on the seat, improved convenience/easier setup of measurement equipment/sensors, reduced interference/discomfort at the user-seat interface.

Several researchers [1,14] have attempted to use infrared thermography in order to avoid the direct contact between body and temperature probes. However, as the subject was required to stand in order to image the surface with the IR camera, accurately determining the thermal changes occurring at the body-seat interface continuously and without being disturbed were not possible. Likewise previous use could not be described as unobtrusive and required a fully mobile subject, rendering its use limited within patients who would be most likely to benefit.

To overcome the aforementioned drawbacks associated with IR imaging, it was decided to determine if it were possible to continuously image the interface through the cushion material with IRs. As the IR signal would be affected by passage through the cushion material, a stochastic digital filter was used to suppress noise at the pre-processing stage. A data-driven algorithm, based on an artificial neural network (ANN), was then used to estimate the contact surface temperatures, compared with data from a temperature sensor placed in the interface. It was also decided to investigate the effect of cushion foam density and thickness, as it was expected that these factors would affect IR transmission.

## 2. Materials and Methods

### 2.1. Participant

An asymptomatic male university student (180 cm and 75 kg), with no history of musculoskeletal problems (e.g., back pain) within three months prior to the experiments, volunteered and consented to take part in the trials. The participant was instructed about the procedures before attending the following trials and was told not to engage in any vigorous activities 24 h ahead of the experiments. Although the study did not require the ethical approval [17], a proposal was submitted to, and permission was granted by the Faculty Research Committee.

### 2.2. Experimental Design

Measurements were performed in a room where ambient temperature and relative humidity (RH) were continually monitored (mean ± SD): 26.8 ± 0.7 °C and 64.6 ± 5.5 %RH. The door of the research room was closed during the trials to reduce any disturbance, either to the experimental environment or to the continuity of the experiments.

To prevent any impact of clothing materials on temperature measurement, the participant was asked to wear cotton pants (preferably the same manufacture and design) on each occasion he attended the laboratory. Before being seated, the participant was asked to empty his bladder to reduce/avoid the need of a break during the sitting period and to prevent discomfort causing changes in seating posture.

The participant was allowed to complete the experiments at his convenience, but was requested to choose a particular time of day which he would be free to attend on different days, in order to limit diurnal variation. During each trial, the participant would sit on a foam cushion for an hour. After each one-hour sitting trial (total number of trials was 32: sitting once on each of the 16 thicknesses for each of the two chosen foam densities), the foam cushion was left to re-equilibrate to room temperature before commencing the next round of tests. Consequently, the trials continued for over a month (from 25 July 2019 to 29 August 2019).

Prior to the beginning of each trial, the participant was asked to stand in front of the seat and wait one minute for the researcher’s instruction to sit down [18]. Whilst seated, the participant was allowed to read books or listen to music, but was asked to maintain an upright sitting posture and not undertake any unnecessary movements (e.g., leg crossing or fidgeting) [18].

### 2.3. Temperature Sensors

#### 2.3.1. Sensor Description

In terms of direct contact temperature measurement (DCTM), DS18B20 (Maxim Integrated, San Jose, CA, USA) was used and its performance was evaluated in the previous research work [19]. In comparison, a commercially available IRs (MLX90614, Melexis Technologies, Tessenderlo, Belgium) was employed for the non-contact temperature measurement (NCTM).

#### 2.3.2. Data Acquisition and Transmission

As the IRs has I^2^C (Inter-Integrated Circuit) bus interface, two specific digital pins of an ATmega328 processor (Microchip Technology, Duluth, MN, USA) were connected to the sensor’s SDA (Serial Data) and SCL (Serial Clock) pins. For the purpose of real-time data transmission and storage, one USB (Universal Serial Bus) port was employed by connecting the computer with the microprocessor via a commercially available circuit board embedded with the FT232R chip (Future Technology Devices International Limited, Glasgow, UK). The sampling frequency was set to 1 Hz, as temperature at the body-seat interface changes slowly [5,6]. Regarding DCTM, DS18B20 was connected to the microprocessor based on the one-wire protocol [19].

#### 2.3.3. Sensor Performance Evaluation

To verify the performance of the IRs, a standardized temperature chamber (PVS-3KP, ESPEC Assist Co., Osaka, Japan) was utilized as the reference source, having the capability of providing reliable outputs ranging from −20 ± 0.5 °C to 100 ± 0.5 °C (Certificate No: ISO 04308Q11746R0 M and EN AC/0708030). In accordance with previous studies in this area [5,6,13], sensor calibration took place by manually adjusting the chamber’s temperature between 20 °C and 50 °C with increment/decrement steps of 5 °C, aiming to assess the sensor’s hysteresis and linearity along with accuracy and reliability. After the chamber’s temperature reached a stable status, consecutive 20-s measurements of the IRs output were recorded and averaged to determine the corresponding output for each pre-set evaluation point. As the DCTM sensor (DS18B20) had been verified in the previous research work [5,19], the related evaluation process was not repeated this time. However, the performance of DCTM sensor was re-evaluated in comparison with the IRs, partly since it has been more than two years away from the last full calibration test.

#### 2.3.4. Sensor Positioning

**Figure 1:**
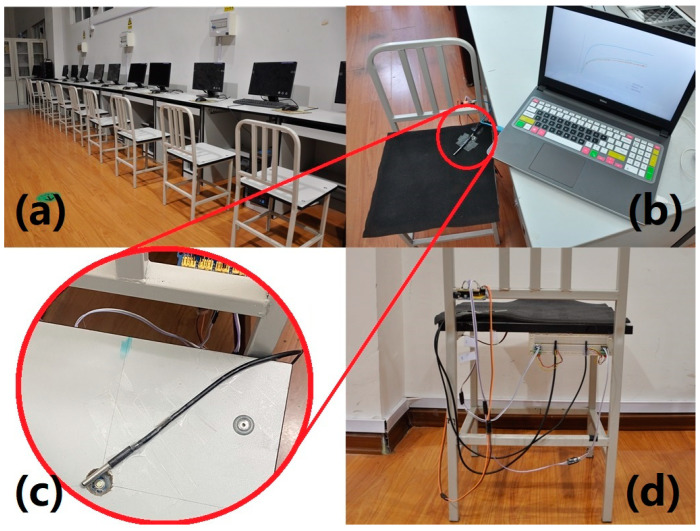
System construction of temperature measurement with temperature probe under the left ischial tuberosity and IRs directly beneath the cushion at the same location: (**a**) a perspective view of the research room with test chairs and computers; (**b**) top view of the experimental chair installed with 18B20 temperature probe (a water-proof metal cylinder which does not appear to significantly affect sitting comfort in asymptomatic users [19]) fastened onto the foam cushion by transparent tape; (**c**) IRs embedded in the wooden chair (to better illustrate the location of the IRs with respect to the temperature probe, the foam cushion has been removed); (**d**) rear view of the chair illustrating the data acquisition and data transmission elements.

The position for temperature measurement was located under the left ischial tuberosity, as previous research indicated this region to be one of the most significant areas for determining thermal changes after prolonged sitting [10,13]. To perform NCTM and DCTM simultaneously, the IRs was held in place using hot melt adhesive (Delixi Electric Ltd., Shanghai, China) to a small hole (1.8 cm in diameter) drilled through the wooden chair (0.5 cm away from the bottom side of the foam cushion) while the temperature probe was placed on the top layer of the stacked foams (Figure 1). Consequently, the DS18B20 temperature probe lay between the body and the foam cushion during the sitting trials while the IRs was underneath the foam cushion.

### 2.4. Foam Cushions

#### 2.4.1. Description

Since foam density has an impact on the thermal conductivity, experiments were conducted using two types of foams commonly used in seating [20,21]: low density (10.8 kg/m^3^) and intermediate density (22.1 kg/m^3^). Cushion thickness is another important factor that was expected to affect thermal conductivity, and therefore a range of different thicknesses of each density of cushion foam were tested. 0.5 cm thick slices of foam were stacked on top of each other to increase the cushion thickness in the range of 0.5 to 8 cm. A wooden chair was used as the frame base to support the foam cushions. Foams (purchased from Taobao Co., Hangzhou, China) were cut it into appropriate pieces (50 × 50 × 0.5 cm: Length, Width and Thickness, respectively) to fit onto the wooden chair’s support surface.

#### 2.4.2. Cushion Selection

To facilitate randomization of thicknesses and density, different thicknesses (0.5 to 8 cm) were numbered 1 to 15 while low and intermediate densities were labeled A and B, respectively (e.g., A1 represents 0.5 cm low dense foam). Paper tags, written in A1 ~ A15 or B1 ~ B15, were put in an opaque envelope in advance. Before conducting a trial, a paper tag was randomly drawn from the opaque envelope and then destroyed. Then the appropriate foam cushion was prepared, the participant was not informed of either the thicknesses or density of the foam prior to the experiments.

### 2.5. Data Processing and Analysis

#### 2.5.1. Pre-Processing

Due to the fact that thermal measurement can be easily influenced by the local environment [10,13,17] (especially for NCTM), the NCTM data were pre-processed by a digital filter based on the stochastic characteristics of empirical mode decomposition (EMD) [22,23,24,25]. The kernel part of EMD is to separate original signals into slow/fast oscillating components. The procedure of using an EMD-based filter has been introduced previously [9] and is summarized below:

(a) Identification of all of the maxima and minima in the original signal;
(b) Interpolate the maxima and minima using a cubic spline function and form upper/lower envelopes. Then divide the summation of the upper and the lower envelopes by two to get the averaged envelope;
(c) Subtract the averaged envelope from the signal and iterate until the averaged envelope approximates to zero. Eventually, a series of intrinsic mode functions (IMFs) and a residue are achieved;
(d) Reconstruct the de-noised signal by adding up the components whose fractal scaling indices (FSI) are greater than the threshold [24,25].

#### 2.5.2. Statistical Analysis

The relationship between DCTM and NCTM was analyzed using the Pearson correlation coefficient. A one-way analysis of variance (ANOVA) was used to examine the influence of different thicknesses on temperature measurement, with significance level set to *p* ≤ 0.01. All analysis was conducted and displayed using Matlab (MathWorks Co., Natick, MA, USA) and Excel (Microsoft Co., Seattle, WA, USA).

#### 2.5.3. Prediction Model

To minimize the impact of thickness, the data-driven ANN model was chosen to estimate the body-seat interface temperature using the de-noised IRs data [26,27]:

(1) $$y=f\left(\sum_{i=1}^{n}\omega_{i}x_{i}+b_{i}\right)$$

where $x_{i}$ represents the input vector, ${\;\omega }_{i}$ is the weighted coefficient, and $b_{i}$ is the bias. The output vector is *y* and *f* is named the transfer function, while *n* is the number of neurons. In our application, three-layered Levenberg-Marquardt back propagation neural networks [28] were employed, including the input, hidden and output layers. In addition, a tangent-sigmoid function was utilised as the transfer function of the hidden layer, which performs a critical role in the optimal operation of the ANN. For the output layer, the linear function was used. The complete training data sets were divided into three parts, with 70% for training, 15% for validation and 15% for testing.

#### 2.5.4. Prediction Evaluation

The data-driven ANN model was evaluated with respect to two statistical criteria: root mean square error (RMSE), Nash-Sutcliff efficiency (NSE) and mean absolute error (MAE). These indicators can be expressed by the following equations [26,27]:

(2) $$\mathrm{RMSE}=\sqrt{\frac{1}{N}\sum_{i=1}^{N}{\left(T_{d}-T_{e}\right)}^{2}}$$

(3) $$\mathrm{NSE}=1-\frac{\sum_{i=1}^{N}{\left(T_{d}-T_{e}\right)}^{2}}{\sum_{i=1}^{N}{\left(T_{d}-\bar{T_{d}}\right)}^{2}}$$

(4) $$\mathrm{MAE}=\frac{\sum_{i=1}^{N}\left|T_{d}-T_{e}\right|}{N}$$

where $T_{d}$ and $T_{e}$ are the DCTM values and the ANN-based estimation using NCTM data, respectively. Additionally, *N* is the number of data sets and ${\bar{T}}_{d}$ is the average value of $T_{d}$. In terms of the associated DCTM, the lower the RMSE (closer to 0) or the higher the NSE (closer to 1) value, the better the estimation it represents.

## 3. Results

### 3.1. Verification of the Sensor Accuracy and Reliability: Ascending/Descending Temperature Challenge Performed in the Controlled Temperature Chamber

**Figure 2:**
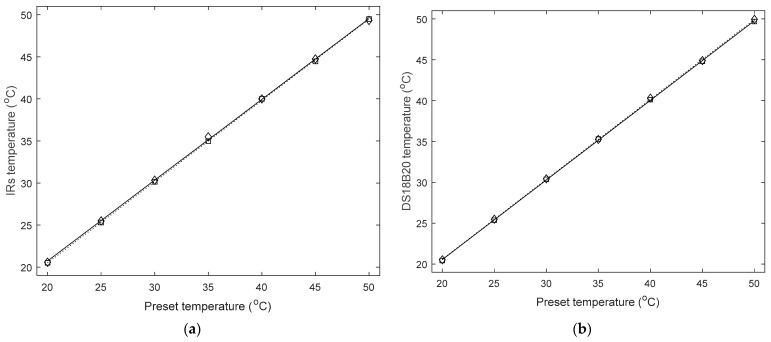
Sensor performance evaluation: (**a**) IRs and (**b**) DCTM sensor (DS18B20). Diamonds “◇” represent the increasing values and squares “□” represent the decreasing values. In addition, the dotted line indicates the trend for the temperature increasing test, while the solid line shows the trend for the temperature decreasing test. The equation of IRs for temperature increase was y = 0.957x + 1.628 whereas that for temperature decrease was y = 0.966x + 1.181. In comparison, the equation of the DCTM sensor for temperature increase was y = 0.965x + 1.218 whereas that for temperature decrease was y = 0.959x + 1.555.

Recordings from the IRs and the DCTM sensor (DS18B20) sampled at 1 Hz were averaged every 20 s (20 recordings) and compared with the pre-set values of the temperature chamber (Figure 2). The maximum absolute error between the averaged measured values and the temperature chamber output was 0.49 °C (IRs) and 0.44 °C (DS18B20) over the whole testing range. Regarding reliability, the maximum standard deviation within the testing range (20 °C to 50 °C ) was 0.05 °C and 0.04 °C.

Output from the IRs also exhibited an approximately linear relationship to that from the standardized temperature chamber (R^2^ = 0.9999 and 0.9997 for temperature increasing and decreasing trials, respectively). In regard to hysteresis, the absolute difference of corresponding points between increment and decrement trials was <0.23 °C. In terms of the DCTM evaluation, R^2^ = 0.9999 for both increment and decrement trials and the absolute difference was <0.20 °C.

### 3.2. Temperature Data Pre-Processing: Application of EMD-FSI Filter

**Figure 3:**
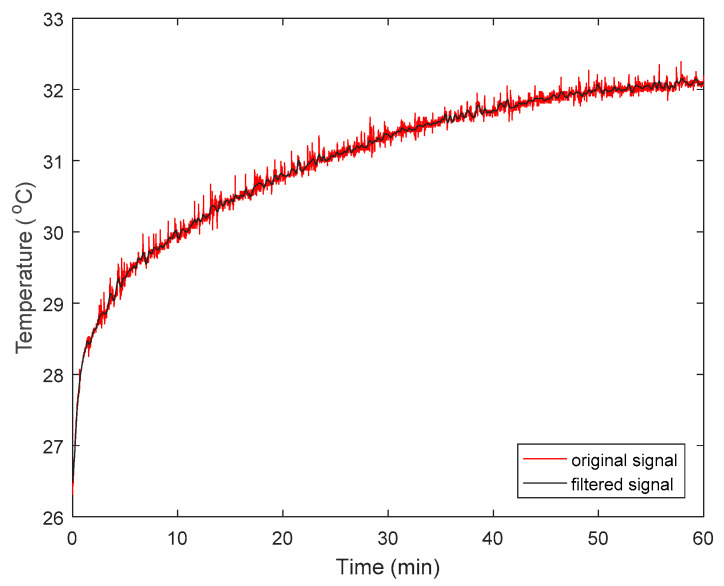
Comparison between the original (red curve) and filtered signals (black curve) with the help of the EMD-FSI noise suppression algorithm. The filtered signal was reconstructed from IMF5 to IMF8 along with the residue, while the rest (IMF1 to IMF4) was discarded in the process of signal reconstruction as their FSI values were less than the threshold.

**Figure 4:**
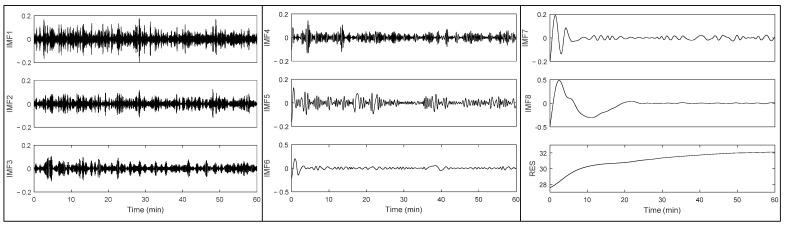
Decomposed components of the original IRs signal. The first eight components (IMF1 to IMF8) were intrinsic mode functions, while the last one (RES) at the bottom right was the residue. The horizontal axis represents the whole sitting period (one-hour epoch of a randomly selected NCTM trial), while the vertical axis represents the temperature values (°C).

We randomly selected a one-hour epoch of NCTM data (low density and thickness = 5 cm) containing spike noises (the red curve in Figure 3) to briefly illustrate the workflow of the EMD-FSI filter. First, the signals were decomposed into eight IMFs and a residue using the EMD algorithm (Figure 4). Then the FSI value of each component was calculated and compared against the threshold (0.5 in our application). As a result, the first four IMFs were removed as their FSI values (0.4462, 0.3064, 0.3760, and 0.3305, respectively) were less than the threshold, while IMF5 to IMF8 and the residue (FSI values: 0.5580, 0.6070, 0.6053, 0.9602, and 1.0050, respectively) were retained to reconstruct the noise-free signal (black curve in Figure 3).

### 3.3. Body-Seat Interface Temperature Estimation: The Relationship between DCTM and NCTM

**Figure 5:**
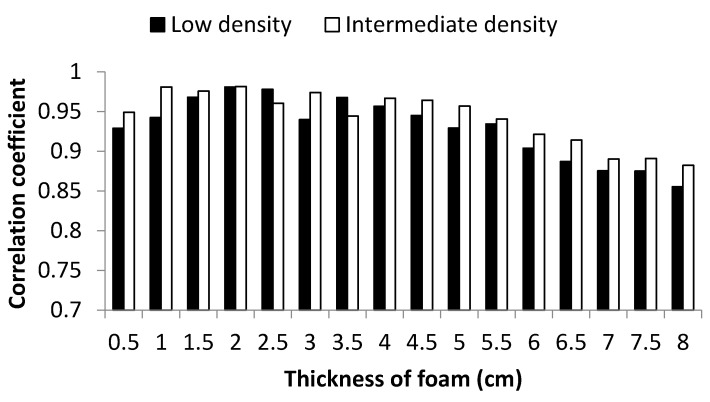
Comparison of Pearson’s correlation coefficients between DCTM and NCTM in relation to thickness of the cushion and the foam density.

The most important first step in effectively determining whether the body-seat iterface temperature can be accurately and reliably determined using an IRs was to investigate the relationship between DCTM and NCTM. Figure 5 shows the Pearson correlation coefficients between the data from DCTM and NCTM for foam cushions in terms of different thickness and density.

**Figure 6:**
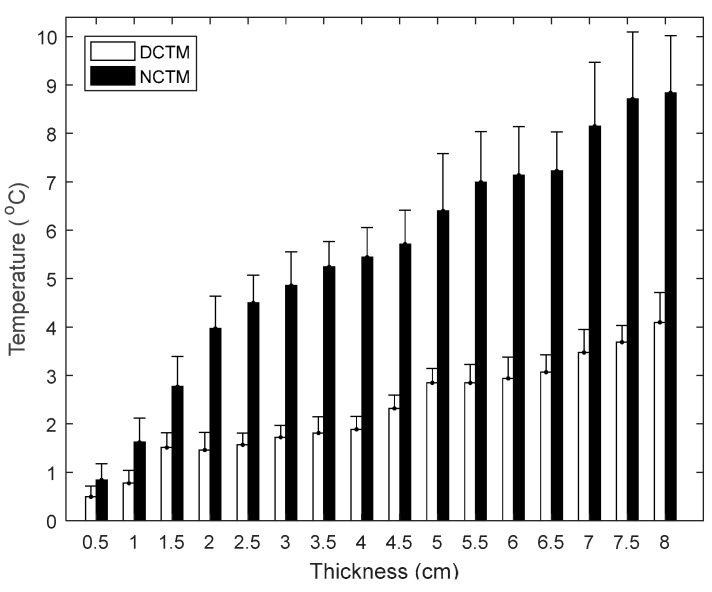
Averaged temperature differences (using one-hour sitting test data) between DCTM and NCTM for low/intermediate density foams regarding different thickness. Error bars denote ±1 standard deviation.

As the thickness increased, the difference in temperature between NCTM and DCTM became more obvious (Figure 6). This was only really noticeable when the thickness was above 6.5 cm, with lower thicknesses having greater correlation coefficients between NCTM and DCTM (ρ > 0.9). In addition, the thermal difference (DCTM-NCTM values) among different thickness for low/intermediate density was significant (ANOVA: *p* < 0.01), with the thermal difference for the low-density foam being much smaller than that for the corresponding thickness of intermediate density foam (0.5 cm thick: 0.49 ± 0.22 °C for low density while 0.84 ± 0.34 °C for intermediate density; 8 cm thick: 4.09 ± 0.62 °C for low density foam while 8.84 ± 1.18 °C for the intermediate density).

**Figure 7:**
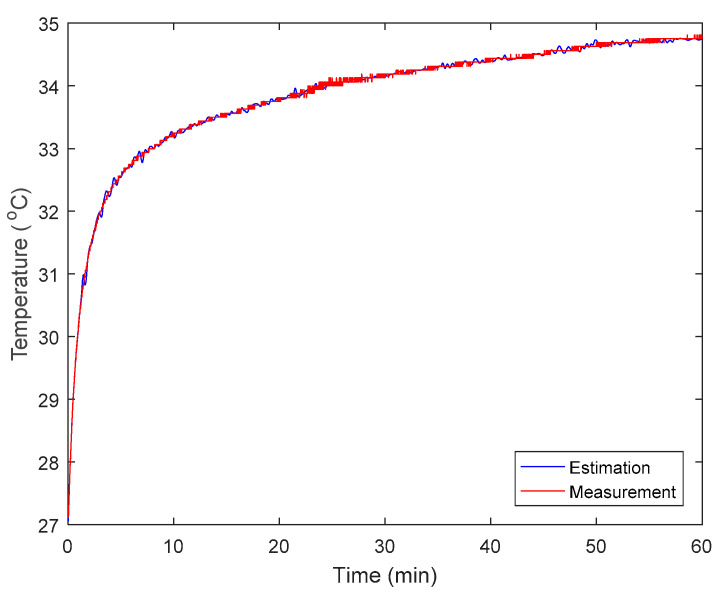
Comparison between DCTM (the red curve referred to as “Measurement” in the graph) and ANN-based estimation (the blue curve referred to as “Estimation” for clarity in the graph) using a randomly selected one-hour NCTM dataset (low density foam with thickness = 5 cm).

A data-driven ANN model was adopted to estimate the body-seat contact surface temperature, in order to minimize the impact of thickness on NCTM. The estimation performance was also evaluated using two indices (Table 2). In addition, the estimated outcomes based on randomly selected NCTM data series were compared with the corresponding DCTM values (Figure 7).

## 4. Discussion

This pilot study is, to the authors’ knowledge, the first to report on measuring continuous body-seat interface temperature comparing contact and non-contact methods. The direct measuring method (“gold standard” in this case) was subjected to rigorous calibration assessments to ensure it was a reliable and accurate reference for use against the IRs combined with an EMD-FSI filter, designed to suppress the noise contained in the original data, and data-driven ANN algorithm, to estimate the contact surface temperature mitigating the impact of foam thickness and density.

### 4.1. IRs Performance

Based on the results of the evaluation trials, it appears reasonable to conclude that the IRs-software solution reported here would be suitable for non-contact body-seat interface temperature measurement. Reasons include an accuracy within 0.5 °C across the detection range (0 °C to 50 °C) according to the sensor’s datasheet (partially verified by the findings in the sensor evaluation stage), and the digital I^2^C peripheral interface. The latter enables the IRs to be directly accessed by most of the currently popular microprocessors without purchasing any auxiliary electronic chips. Beyond that, the size of the sensor is sufficiently small (1.1 cm in diameter) to be integrated into an electronic circuit board. As the price of a single IRs was approximately 3.5 USD (at the time of the experiment), cost of configuring a detection matrix to measure the whole body-seat interface would not be commercially prohibitive.

### 4.2. Noise Suppression Algorithm

The EMD-FSI filter was applied to the raw IRs data before carrying out further analysis, as the IRs output may be affected by a varying ambient environment. The EMD-FSI filter behaves as a dyadic filter bank resembling those observed in classical wavelet decomposition [23,24]. Due to its characteristics, there is no need to specify central frequencies or bandwidths when performing filtering operations. In addition, the reconstruction criterion is fully adaptive as it stems from the stochastic analysis of random noises [23].

### 4.3. Impact Factors on Temperature Estimation

All correlation coefficients between NCTM and DCTM were >0.85 (*p* < 0.01), which indicated there to be a strong relationship between the contact and non-contact measurements when used in this way. Expectedly, the correlation coefficient value decreased as the thickness increased, becoming especially noticeable with thicknesses >6.5 cm. This appears to be mainly the result of a greater attenuation of the thermal conductivity with increasing foam thickness [29].

Another factor affecting the accuracy of the data-driven algorithm appears to be the density of the foam. Using the intermediate density foam with a thickness of 6.5 cm as an example, RMSE values increased by 20%, 40%, and 60% for thicknesses of 7 cm, 7.5 cm, and 8 cm, respectively. In comparison, there was less change for low density foam at the corresponding thickness (0%, 17%, and 17% increment for 7 cm, 7.5 cm, and 8 cm by taking 6.5 cm as the reference, respectively). The RMSE value (Mean ± SD) for low density foam was 0.06 ± 0.01 °C when comparing the direct measurement with the estimated values, while the equivalent RMSE value for the intermediate density foam was 0.09 ± 0.03 °C. In addition, MAE values for low/intermediate density foam are less than 0.05 °C and 0.09 °C, respectively. This further illustrates the robustness of the estimation algorithm.

As the RMSE values of both low and intermediate density foams were close to zero, the data-driven ANN algorithm proved effective at overcoming the impact of different densities on thermal conductivity. Furthermore, all NSE values were >0.99 which indicated the algorithm to be useful when attempting to estimate the body-seat interface temperature using the IRs system.

Other aspects that should be considered when measuring the thermal changes at the body-seat interface include air flow speed, mean radiant temperature (MRT), and sweating. To reduce the impacts of these factors, we conducted the whole experiments in a controlled research room with the closed door and continuously monitored the ambient temperature and relative humidity. Although MRT is an element of subjective thermal comfort [30,31], the foam cushion was left to re-equilibrate to room temperature before commencing the next round of tests, which can effectively reduce the impact of MRT on thermal perception of the participant.

### 4.4. Limitations

Although potentially useful findings have been reported here, there are still several limitations in the current study. Firstly, the data were generated from a single subject. Although this could be considered appropriate for generating data for a simple study such as this, designed to verify the possibility of using IRs in this role, the approach cannot be extrapolated to the broader population without widening data collection to include participants with greater anthropometric variation, age, sex, and even degrees of infirmity. Future trials would, therefore, need to involve a larger variation and number of participants so that a more general estimation model could be developed by taking into account such variables as well as other relevant aspects: clothing and environment (e.g., temperature and relative humidity) [13]. Furthermore, feature ranking is needed to be considered, when conducting future experiments, which includes different participants (gender, body mass, height) and various experimental conditions (cushion materials, room temperature and relative humidity). This said, the results of this study have confirmed the feasibility of continuously approximating temperature at the body-seat interface without direct contact. Use of the approach outlined here would effectively reduce the sitting discomfort caused by the presence of temperature probes, enable long term monitoring of temperature at the body-seat interface, facilitate the development of whole seat surface monitoring systems and avoid the need for intermittent standing from chairs in comparison to thermographic measurements [1,14]. Such a change would be expected to benefit researchers, seat designers and potentially clinicians and carers (e.g., those responsible for care of some wheelchair users and dementia sufferers). In relation to the potential importance for clinicians and carers, it has been noted previously that obtrusive temperature probes can exacerbate formation of cutaneous lesions on wheelchair users [10]; furthermore, frequently lifting disabled people from wheelchairs to perform thermographic measurement is challenging and impractical in real life [32]. The need to consider wheelchair users is one of our future aims, which cannot be achieved until we can verify the ability to use IR in this situation and determine its limitations.

Though this study compared two typical foams (low/intermediate density) widely used by upholstery manufacturers [29], it is necessary to widen the investigation to include various other densities/thicknesses and even formulations of seating material (e.g., gel inserts) in order to determine both feasibility and optimal thickness for IRs recording in each case. Additionally, there is the need to determine where the performance of the proposed method deteriorates beyond being reliable, as may be the case for some of the very high-density foams or gels used in specialized seating applications. However we have not yet tested this.

Since the IRs was not in contact with the measured object, it was easier to embed it into the chair frame, which also serves to limit any disturbance to the sensor and its circuitry caused by the surrounding environment. Although the use of ANN has been reported to outperform several methods [26,27] and appeared suitable for this study, a thorough investigation of possible alternatives was not made.

Notwithstanding the above limitations, the authors consider that the findings presented here significantly contribute to the scientific research and industrial application of thermal measurement at the body-seat contact surface by showing that an estimation of temperature which is highly comparable with that from contact sensors at the interface is possible by combining the non-contact thermal detection technique with a data-driven algorithm. Therefore, these findings should provide researchers/seat designers and clinicians with less obtrusive measurement options that appear to be equally reliable methods of objectively studying thermal comfort of seats without interfering with the activities of the users. As a result, these findings would support future development of effective thermal regulation and even automated skin ulcer prevention protocols for seats, which could be instrumental in reducing the potential for tissue damage during prolonged sitting.

## 5. Conclusions

A NCTM system was developed using an IRs-software system, to determine if such a method could be reliably used in the continuous monitoring the thermal changes at the body-seat interface. Strong correlations (ρ > 0.85) were found between non-contact measurements and those produced by a contact thermal probe placed directly in the interface space (the “gold standard” [10,16,17]). To reduce the potential attenuating effects of thickness and density of the foam on thermal conductivity, an ANN-based data driven algorithm was used to estimate the thermal changes, resulting in RMSE close to zero and NSE ≈ 1. Based on the experimental results, NCTM appears to provide a promising tool for unobtrusive continuous measurement of thermal changes at the body-seat interface in the future.

## Figures and Tables

**Table 1:** Comparison of methods measuring the body-seat interface thermal characteristics.

Methods	Tools	Advantage	Disadvantage
Subjective	Questionnaires [15]	Straight-forward implementation	Requires a large number of populations and liable to be influenced by subjective factors (e.g., mood and aesthetics).
Objective	Temperature probes [5,6,13]	Continuous and real-time measurement	Damage to the integrity of the cushion by embedding probes or perceptible if attached to the body.
	Thermography [1,14]	Whole seat thermal information	Measure discontinuously and require sitters to stand for thermal images acquisition

**Table 2:** Evaluation of the data driven ANN-based estimation.

Thickness (cm)	Low Density Foam			Intermediate Density Foam		
	RMSE (°C)	MAE (°C)	NSE	RMSE (°C)	MAE (°C)	NSE
0.5	0.06	0.04	0.9943	0.05	0.04	0.9995
1.0	0.06	0.04	0.9952	0.07	0.05	0.9990
1.5	0.06	0.04	0.9974	0.07	0.05	0.9984
2.0	0.05	0.03	0.9982	0.06	0.04	0.9989
2.5	0.06	0.04	0.9914	0.10	0.06	0.9969
3.0	0.07	0.05	0.9950	0.10	0.06	0.9971
3.5	0.05	0.03	0.9919	0.06	0.04	0.9987
4.0	0.05	0.03	0.9938	0.10	0.06	0.9974
4.5	0.04	0.03	0.9961	0.07	0.05	0.9985
5.0	0.07	0.05	0.9927	0.11	0.06	0.9979
5.5	0.06	0.04	0.9932	0.08	0.05	0.9986
6.0	0.04	0.03	0.9961	0.10	0.06	0.9980
6.5	0.06	0.04	0.9869	0.10	0.06	0.9965
7.0	0.06	0.04	0.9945	0.12	0.07	0.9981
7.5	0.07	0.05	0.9970	0.14	0.08	0.9955
8.0	0.07	0.05	0.9985	0.16	0.09	0.9967

## Data Availability

The data presented in this study are available on request from the corresponding author.
